# Peptidoglycan Contribution to the B Cell Superantigen Activity of Staphylococcal Protein A

**DOI:** 10.1128/mBio.00039-21

**Published:** 2021-04-20

**Authors:** Miaomiao Shi, Stephanie E. Willing, Hwan Keun Kim, Olaf Schneewind, Dominique Missiakas

**Affiliations:** a Howard Taylor Ricketts Laboratory, Argonne National Laboratory, Lemont, Illinois, USA; b Department of Microbiology, University of Chicago, Chicago, Illinois, USA

**Keywords:** B cell receptor, LysM, protein A, *Staphylococcus aureus*, peptidoglycan, superantigens

## Abstract

Staphylococcus aureus causes reiterative and chronic persistent infections. This can be explained by the formidable ability of this pathogen to escape immune surveillance mechanisms. Cells of S. aureus display the abundant staphylococcal protein A (SpA). SpA binds to immunoglobulin (Ig) molecules and coats the bacterial surface to prevent phagocytic uptake. SpA also binds and cross-links variable heavy 3 (V_H_3) idiotype (IgM) B cell receptors, promoting B cell expansion and the secretion of nonspecific V_H_3-IgM via a mechanism requiring CD4^+^ T cell help. SpA binding to antibodies is mediated by the N-terminal Ig-binding domains (IgBDs). The so-called region X, uncharacterized LysM domain, and C-terminal LPXTG sorting signal for peptidoglycan attachment complete the linear structure of the protein. Here, we report that both the LysM domain and the LPXTG motif sorting signal are required for the B cell superantigen activity of SpA in a mouse model of infection. SpA molecules purified from staphylococcal cultures are sufficient to exert B cell superantigen activity and promote immunoglobulin secretion as long as they carry intact LysM and LPXTG motif domains with bound peptidoglycan fragments. The LysM domain binds the glycan chains of peptidoglycan fragments, whereas the LPXTG motif is covalently linked to wall peptides lacking glycan. These findings emphasize the complexity of SpA interactions with B cell receptors.

## Abstract (precis)

The LysM domain is found in all kingdoms of life. While their function in mammals is not known, LysM domains of bacteria and their phage parasites are associated with enzymes that cleave or remodel peptidoglycan.

## INTRODUCTION

All clinical Staphylococcus aureus isolates elaborate staphylococcal protein A (SpA), a surface protein well known for its ability to bind host immunoglobulins (Igs) (1, 2). SpA is comprised of five immunoglobulin binding domains (IgBDs) (3, 4). Each IgBD assumes a triple-helical structure with a binding site for the Fcγ domain of IgG (5). Interaction with Fcγ prevents C1q binding and complement activation, effectively blocking a critical effector function of antibodies (6, 7). Earlier work also demonstrated that S. aureus stimulates the proliferation of isolated human B cells in a manner that requires SpA (8, 9). Purified SpA extracted from the bacterial envelope was shown to promote B cell proliferation in mice and human lymphocyte preparations in the presence of CD4^+^ T helper cells (10–12). This mitogenic activity is facilitated by a second site of binding of the IgBDs to the conserved surface of Ig antigen-binding fragment (Fab), which is encoded by variable heavy 3 (V_H_3) clan-related genes (13). SpA can cross-link the V_H_3-IgM B cell receptors (BCRs) to trigger supraclonal proliferation of B cells (9, 11). During infection of mice and humans, B cell superantigen activity leads to the expansion of V_H_3 idiotype plasmablasts in blood and to the secretion of V_H_3 idiotype antibodies that lack recognition of staphylococcal antigens (12, 14). In this manner, SpA may contribute to the lifelong association of S. aureus with humans and the bacterium’s ability to cause reiterative infections (15).

SpA is synthesized as a precursor in the bacterial cytoplasm and enters the secretory pathway via an N-terminal signal peptide (16). Once translocated across the plasma membrane, the signal peptide is cleaved by signal peptidase and sortase A (SrtA) cleaves the C-terminal LPXTG sorting signal after threonine (T) and transfers the processed polypeptide to the terminal glycine (G) residue of the peptidoglycan intermediate [C_55_-(PO_4_)_2_-MurNAc(l-Ala-d-iGlu-l-Lys(NH_2_-Gly_5_)-d-Ala-d-Ala)-GlcNAc] known as lipid II (17, 18). The SpA-lipid II intermediate is then incorporated into cell wall peptidoglycan and displayed on the bacterial surface (19). During cell division, staphylococci release a portion of their peptidoglycan and peptidoglycan-linked SpA into the extracellular milieu, owing to muralytic enzymes that degrade the cell wall in the vicinity of septal membranes (20–22). Released SpA is tethered to wall peptide with the C-terminal structure l-Ala-d-iGln-l-Lys(SpA-LPET-Gly_5_)-d-Ala-Gly_4_ (23). Native SpA released from the staphylococcal cell wall, but not recombinant SpA purified from the cytoplasm of Escherichia coli, is sufficient to induce the expansion of V_H_3 idiotype IgG and IgM in mice (12).

In this study, we asked whether SpA modification with peptidoglycan is necessary for the expansion of V_H_3 clonal immunoglobulin. By engineering strains that carry the *spa* gene lacking defined domains of the protein, we observed that S. aureus *spa*_ΔLPXTG_, a variant that can no longer immobilize SpA in the envelope, fails to exhibit B cell superantigen activity *in vivo* and *in vitro*. Further, the predicted LysM domain of SpA, positioned between the envelope spanning region X (Xr) and the LPXTG sorting signal, also contributes to B cell superantigen activity by binding the glycan chains of peptidoglycan fragments. Thus, together with peptidoglycan linked to the LPXTG motif, the LysM domain activates immune cells to implement the B cell superantigen activity of staphylococcal protein A.

## RESULTS

### S. aureus *spa* variants lacking defined C-terminal subdomains of protein A.

**FIG 1:**
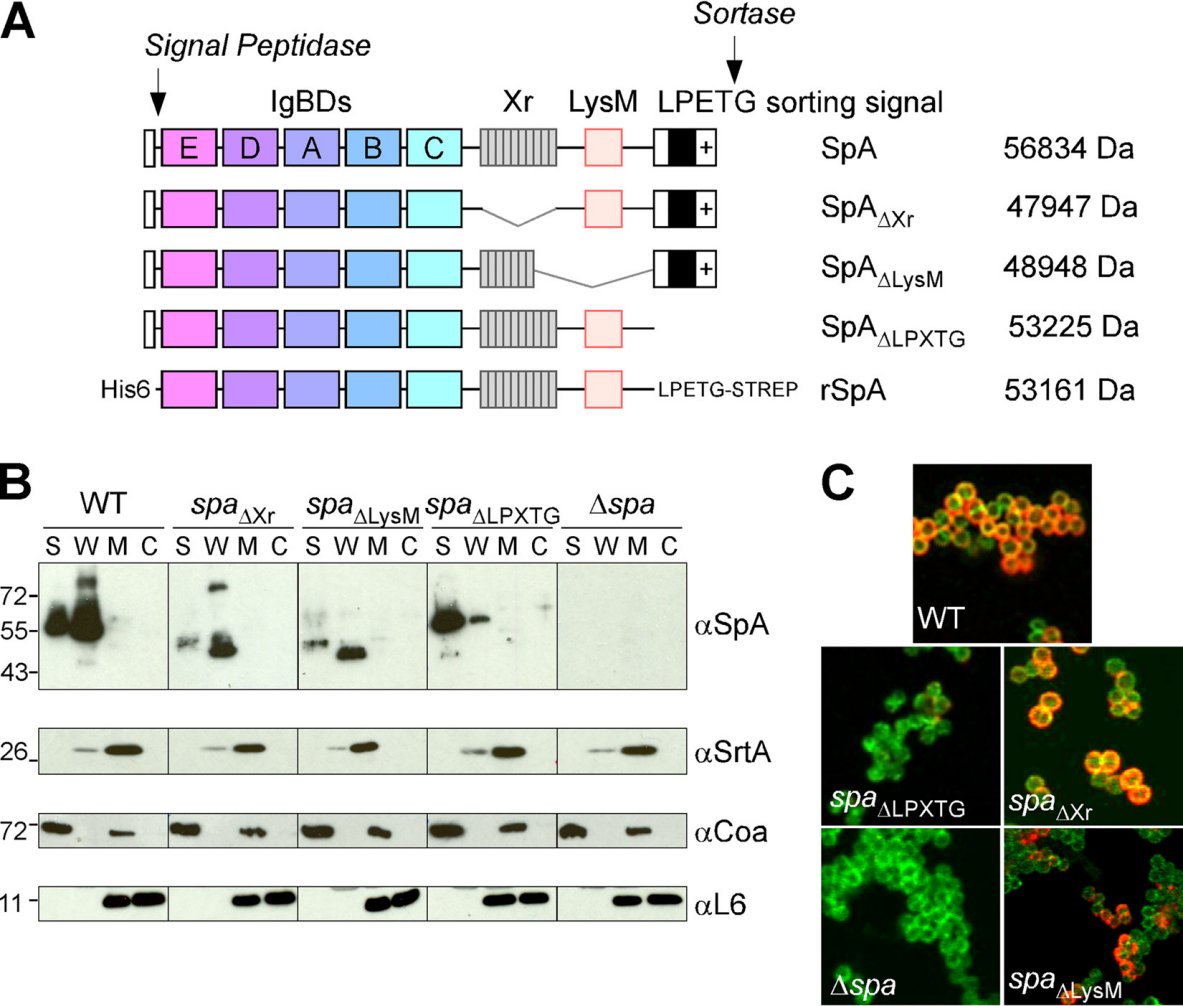
C-terminal domains of staphylococcal protein A (SpA) and their impact on the subcellular localization of SpA variants. (A) Diagram illustrating the structure of the primary SpA translation products with a N-terminal signal peptide (white rectangle) and signal peptidase cleavage site, five immunoglobulin binding domains (IgBDs) designated E, D, A, B, and C, region X (Xr) with 10 repeats of an octapeptide, predicted LysM domain, and C-terminal sorting signal cleaved by sortase between the threonine (T) and the glycine (G) of its LPXTG motif. Variants SpA_ΔXr_, SpA_ΔLysM_, and SpA_ΔLPXTG_ lack amino acid sequences encompassing the Xr, LysM domain, and LPXTG sorting signal as illustrated. rSpA depicts recombinant SpA produced in E. coli without a signal sequence and sorting motif. Molecular weights corresponding to the primary sequence of proteins are shown. (B) Fractionation of S. aureus cultures into culture supernatant (S), lysostaphin-digested cell wall (W), membrane (M), and cytoplasm (C) and immunoblot analysis for the distribution of wild-type and mutant SpA. Antibodies against sortase, coagulase, and ribosomal protein L6 were used to control for successful fractionation. (C) BODIPY-vancomycin (green) staining of peptidoglycan and Alexa Fluor 647-labeled secondary antibody (red) detection of SpA monoclonal antibody 3F6 binding to S. aureus were used to visualize the surface display of SpA.

The translational product of the S. aureus Newman *spa* gene encompasses an N-terminal signal peptide, five IgBDs of 58 to 61 residues (designated E, D, A, B, and C), the so-called region X (Xr) with 10 tandem repeats of the octapeptide EDNNKPGK, a predicted LysM domain, and a C-terminal LPXTG sorting signal (Fig. 1A). Earlier work demonstrated that staphylococcal secretion of SpA encompassing four or five IgBDs is essential for B cell superantigen activity (12). To investigate the contribution of the C-terminal domains of SpA to B cell superantigen activity, we generated S. aureus Newman variants with modified *spa* genes that lacked coding sequences for either Xr (*spa*_ΔXr_), LysM (*spa*_ΔLysM_), or the LPXTG sorting signal (*spa*_ΔLPXTG_) (Fig. 1A). Production of the new SpA variants was assessed by immunoblotting extracts derived from cultures of bacteria fractionated into supernatant (S), cell wall (W), membrane (M), and cytoplasm (C). The S. aureus Newman Δ*spa* strain, which does not produce SpA, was used as a control. Full-length SpA and SpA_ΔXr_ and SpA_LysM_, lacking the C-terminal Xr and LysM domains, respectively, displayed similar fractionation profiles, with most of the proteins found in the cell wall (Fig. 1B). SpA is released from the bacterial peptidoglycan via the combined activities of the murein hydrolases AtlA, Sle, LytM, and LytN (23). Thus, SpA proteins were also found in the culture supernatant (Fig. 1B). SpA_ΔLPXTG_, which lacks peptidoglycan anchoring (LPET-COOH), was predominantly found in the culture supernatant (Fig. 1B). While similar between all mutants, total immune-reactive signals for SpA were reduced compared to those for wild-type bacteria. We attribute this difference to the reduced stability of proteins following secretion in the culture medium. In agreement with this notion, a representative immunoprecipitation experiment shows similar levels of production of SpA and SpA_LysM_ (see Fig. S1 in the supplemental material). Immunoblotting with antibodies against the membrane protein sortase A (anti-SrtA), secreted coagulase (anti-Coa) and cytoplasmic ribosomal protein L6 (anti-L6) was used to validate the fractionation of cultures (Fig. 1B). To further visualize the surface display of SpA, microscopy images of bacteria were acquired using monoclonal antibody 3F6, which binds to the IgBDs of SpA, and Alexa Fluor 647-labeled goat anti-mouse IgG (red) (24). BODIPY-vancomycin (green) was used to visualize peptidoglycan. Cells of the wild type and *spa*_ΔXr_ and *spa*_ΔLysM_ variants displayed SpA on the surface. As expected, cells of the Δ*spa* or *spa*_ΔLPXTG_ mutant could not be labeled with 3F6 (Fig. 1C).

10.1128/mBio.00039-21.2 FIG S1 Rate of synthesis of SpA and SpA_ΔLysM_. S. aureus cultures were pulse-labeled for 60 s with [^35^S]methionine-cysteine. Labeling was quenched by adding an excess of nonradioactive amino acids (chase) for 1 min. After the chase, culture aliquots were precipitated with trichloroacetic acid (TCA), lysostaphin treated, immunoprecipitated with anti-SpA, and analyzed by autoradiography. The experiment was performed in triplicate. Numbers to the left of the autoradiogram indicate the sizes of molecular weight markers in kilodaltons. The slower- and faster-migrating species correspond to precursor and mature SpA and SpA_ΔLysM_, respectively. Owing to the truncation, precursor and mature SpA_ΔLysM_ migrate faster than full-length SpA. Download FIG S1, DOCX file, 0.4 MB. Copyright © 2021 Shi et al. 2021 Shi et al. https://creativecommons.org/licenses/by/4.0/ This content is distributed under the terms of the Creative Commons Attribution 4.0 International license.

### SpA variant-mediated B cell superantigen activity during mouse infection.

**FIG 2:**
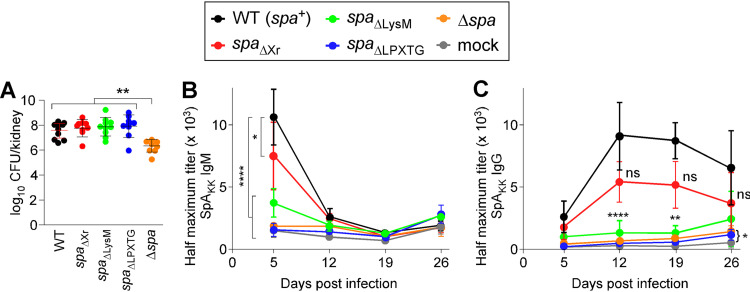
The LPXTG motif and LysM domain of SpA are required for S. aureus B cell superantigen activity in mice. (A) Cohorts of BALB/c mice (*n *= 10) were infected intravenously with 1 × 10^7^ CFU of the S. aureus Newman wild type (WT) or *spa* variant strains. Five days postinfection, kidneys were removed and ground for enumeration of CFU per gram of tissue. (B and C) Cohorts of BALB/c mice (*n *= 10) were infected via intravenous injection with 1 × 10^7^ CFU of S. aureus or a control (PBS [mock]). On days 5, 12, 19, and 26, serum was collected via retro-orbital bleeding and IgM (B) and IgG (C) responses were quantified by ELISA using SpA_KK_, a SpA variant that cannot bind IgG Fcγ but retains the ability to bind V_H_3 variant heavy chains of IgG and IgM. Statistical analysis was performed with one-way analysis of variance (ANOVA) with Dunnett’s multiple-comparison test. ****, *P < *0.0001; ***, *P < *0.001; **, *P < *0.01; *, *P < *0.05. ns, not significant.

Intravenous inoculation of mice with a sublethal dose of S. aureus Newman (1 × 10^7^ CFU) causes bacteremia and persistent abscess formation in all tissues of infected animals (25). During infection, V_H_3 clan IgM and IgG antibodies are produced in a SpA-dependent manner (26). We wondered whether mice infected with S. aureus *spa*_ΔXr_, *spa*_ΔLysM_, *spa*_ΔLPXTG_, and Δ*spa* variants continue to expand V_H_3 clonal antibodies. Because the variants produced less SpA protein, we first compared the abilities of the new strains to disseminate and replicate in mouse kidneys following intravenous infection. At day 5 postinfection, only animals infected with the Δ*spa* variant displayed a significant reduction in bacterial loads in kidney tissues (Fig. 2A). Next, cohorts of animals were infected with the same strains; blood was collected on days 5, 12, 19, and 26 postinfection. Serum samples were subjected to enzyme-linked immunosorbent assay (ELISA) with SpA_KK_, a protein A variant that binds V_H_3 variant heavy chains but not Ig Fcγ (12). Compared to mock-infected animals, mice infected with wild-type S. aureus exhibited a large expansion of V_H_3 idiotype IgM in blood on day 5 postinfection (Fig. 2B). V_H_3 idiotype IgM expansion abated by day 12 and was accompanied by a rise in V_H_3 idiotype IgG sustained over 26 days (Fig. 2C). As expected, animals infected with the Δ*spa* mutant did not display B cell superantigen activity (Fig. 2B and C). Although slightly reduced compared to the case with the wild type, V_H_3 antibody expansions were also observed in animals infected with the *spa*_ΔXr_ variant. Infection of mice with S. aureus *spa*_ΔLysM_ caused a modest expansion of V_H_3 idiotype IgM on day 5, but unlike the case with the wild-type strain or the *spa*_ΔXr_ variant, failed to activate V_H_3 idiotype IgG expansion on day 12 (Fig. 2B and C). Animals infected with the *spa*_ΔLPXTG_ mutant did not display any B cell superantigen activity, corroborating the notion that peptidoglycan modification of naturally released SpA is key to its B cell superantigen activity (Fig. 2B and C). The data suggest that the LysM domain also contributes to SpA-induced expansions of V_H_3 idiotype immunoglobulins during infection.

### Human B cell proliferation induced by S. aureus *spa* variants.

**FIG 3:**
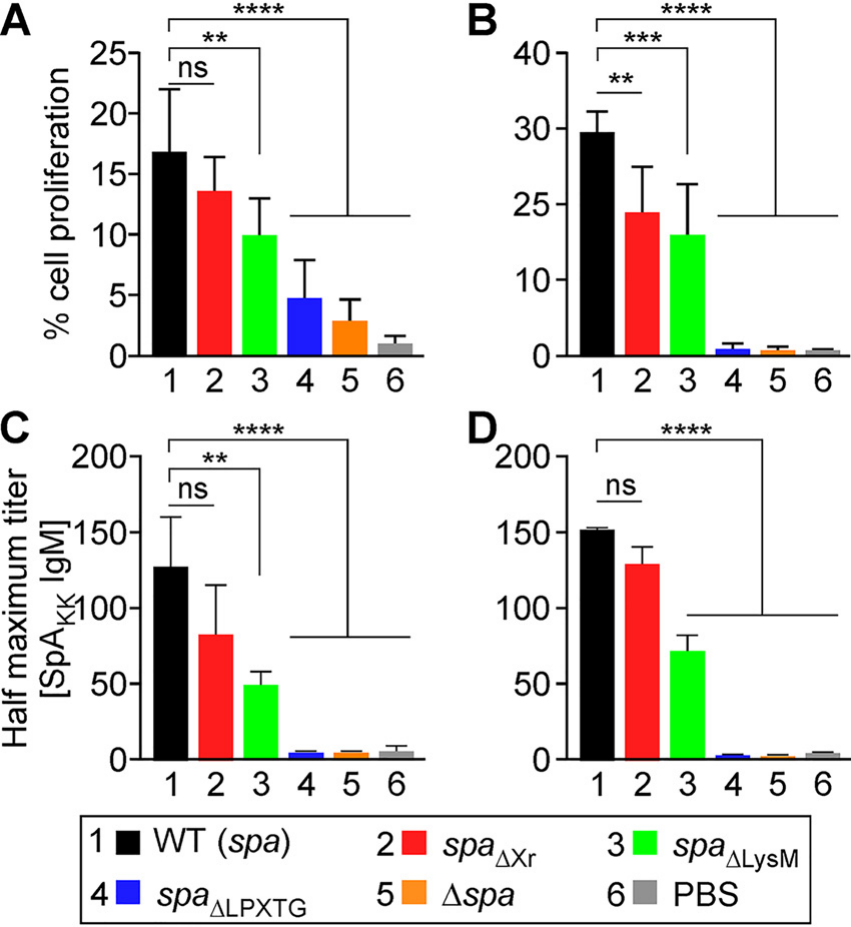
Human B cell proliferation stimulated by the S. aureus Newman wild type and its *spa* variants. (A and B) Proliferation of B cells after stimulation with bacteria. Proliferation of B cells isolated by negative (CD19^+^/CD3^+^ cells [A]) or positive (CD19^+^ cells [B]) selection was calculated via the diluted CFSE signal as shown in Fig. S2C. Bacteria were killed by incubation with 0.5% formaldehyde for 3 h at room temperature, heat treated at 80°C for 3 min, washed, and suspended PBS to equal density. (C and D) Secretion of IgM by B cells (CD19^+^/CD3^+^ cells [C] and D19^+^ cells [D]) after stimulation with bacteria was analyzed by ELISA against SpA_KK_. Data are the means (±SEM) of experimental replicates (*n *= 3 or 4) using B cell preparations from different human donors. Statistical analysis was performed with one-way ANOVA with Dunnett’s multiple-comparison test. ****, *P < *0.0001; ***, *P < *0.001; **, *P < *0.01.

**FIG 4:**
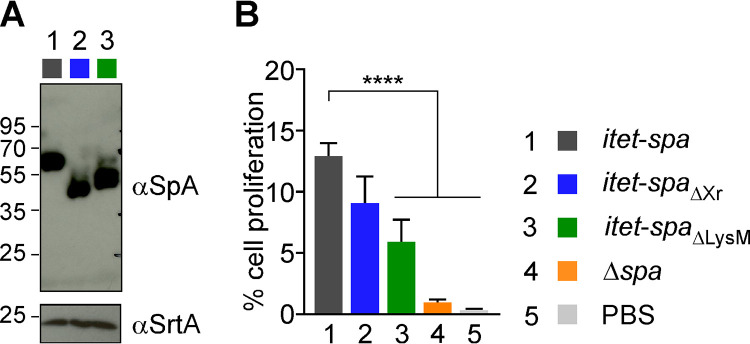
Human B cell proliferation stimulated by *itet-spa* and *itet-spa*_ΔLysM_ strain variants of S. aureus. (A) Cultures of *itet-spa* and *itet-spa*_ΔLysM_ strains were induced with anhydrotetracycline to produce similar amounts of SpA and SpA_ΔLysM_ as shown by Western blotting of lysed cultures. A blot against sortase (αSrtA) documents that a similar number of cells was used to compare the two bacterial variants. (B) Proliferation of B cells isolated by negative (CD19^+^/CD3^+^ cells) selection after stimulation with fixed bacteria grown as described for panel A. Proliferation was calculated via the diluted CFSE signal as shown in Fig. S2. Bacteria were killed by incubation with 0.5% formaldehyde for 3 h at room temperature and heat treated at 80°C for 3 min, washed, and suspended PBS to equal density. Data are the means (±SEM) of experimental replicates (*n *= 3) using B cell preparations from different human donors. Statistical analysis was performed with one-way ANOVA with Dunnett’s multiple-comparison test. ****, *P < *0.0001; ***, *P < *0.001; **, *P < *0.01.

Previous work reported that heat-killed S. aureus cells or staphylococcal extracts induce *in vitro* proliferation of isolated human B cells in a T cell-independent manner (8, 27). To analyze the contribution of specific domains of SpA in this context, S. aureus Newman or its *spa*_ΔXr_, *spa*_ΔLysM_, *spa*_ΔLPXTG_, and Δ*spa* variants were fixed with 0.5% formaldehyde, heat killed, washed in buffer, and added to B cells isolated from human blood mononuclear cell preparations using negative selection (Fig. S2A) or positive selection (Fig. S2B). Proliferation of carboxyfluorescein succinimidyl ester (CFSE)-stained CD19^+^ cells was quantified by measuring dilution of fluorescence over 6 days (Fig. S2C). Compared to mock-treated lymphocytes, incubation of mixed populations of CD19^+^ B cells/CD3^+^ T cells or purified CD19^+^ B cells with formalin-fixed S. aureus Newman at a ratio of 16 bacteria/cell caused proliferation of 16.93% ± 5.16% and 29.36% ± 2.94% of CD19^+^ B cells, respectively (Fig. 3A and B). In contrast, incubation of B cells with the Δ*spa* mutant did not activate proliferation compared to that with phosphate-buffered saline (PBS) (Fig. 3A and B). Incubation of CD19^+^ B cells/CD3^+^ T cells with S. aureus *spa*_ΔXr_ caused proliferation of CD19^+^ B cells in a manner similar to that of wild-type staphylococci (Fig. 3A). However, a small proliferation reduction was observed when S. aureus *spa*_ΔXr_ was incubated with purified CD19^+^ B cells (Fig. 3B). This difference may represent a small contribution of the Xr domain or reflect heterogeneity in blood donors. Incubation with formalin-fixed *spa*_ΔLPXTG_ or Δ*spa* bacteria significantly reduced CD19^+^ B cell proliferation (Fig. 3A and B). This is as expected since SpA_ΔLPXTG_ is no longer retained in the envelope of bacteria and thus is washed away during the preparation of fixed cells. Interestingly, CD19^+^ B cell proliferation was also significantly reduced upon incubation with *spa*_ΔLysM_ fixed bacteria (Fig. 3A and B). To ascertain that the phenotype was not the result of reduced display of SpA_LysM_, three new strains were generated whereby the wild-type *spa*, *spa*_ΔXr_, and *spa*_ΔLysM_ genes were expressed under the control of the tetracycline-inducible promoter. The new strains, the *itet-spa*, *itet-spa*_ΔLXr_, and *itet-spa*_ΔLysM_ variants, were grown to similar densities with adjusted concentrations of inducer to produce similar amounts of SpA proteins (Fig. 4A). Incubation of CD19^+^ B cells/CD3^+^ T cells with the *itet-spa*_ΔLysM_ strain resulted in reduced expansion of B cells compared to that with *itet-spa* (isogenic wild-type) S. aureus (Fig. 4B), confirming that the LysM domain contributes to SpA activity.

10.1128/mBio.00039-21.3FIG S2CD4^+^/CD8^+^ T lymphocyte ratio in human B cell preparations. (A and B) Populations of CD19^+^ B cells and CD3^+^ T cells were quantified following depletion of human blood mononuclear cells with biotinylated antibodies against CD2, CD14, CD16, CD36, CD43, and CD235a and anti-biotin microbeads and exclusion of dead cells via Hoechst 33258 staining. (C) Histogram plot of the representative CFSE fluorescence of CD19^+^ B cell population after 6-day stimulation with killed S. aureus. Mock (PBS)-treated B cells did not dilute CFSE signals (undivided population in gray), whereas the S. aureus WT induced 1, 2, 3, 4, or 5 cell divisions (black) after 6 days. Proliferation in percent was calculated with the equation $\sum_{1}^{i}\frac{N_{i}}{2^{i}}/\sum_{0}^{i}\frac{N_{i}}{2^{i}}$ (*i* is the division number and *N_i_* is the cell number in division *i*). Autofluorescent unlabeled CD19^+^ B cells are traced in blue. Download FIG S2, DOCX file, 2.3 MB.Copyright © 2021 Shi et al.2021Shi et al.https://creativecommons.org/licenses/by/4.0/This content is distributed under the terms of the Creative Commons Attribution 4.0 International license.

As observed with animal infection with live bacteria, the cell expansion of human lymphocytes upon incubation of fixed S. aureus Newman or its *spa*_ΔXr_ variant resulted in robust IgM secretion; this was not observed with the *spa*_ΔLPXTG_ and Δ*spa* variants (Fig. 3C). S. aureus activation of IgM secretion by CD19^+^ B cells did not require CD3^+^ T cells, as similar amounts of immunoglobulin secretion were observed when S. aureus Newman or its *spa*_ΔXr_ variant were incubated with purified CD19^+^ B cells (Fig. 3CD). Thus, whole-cell S. aureus preparations activate CD19^+^ B cell proliferation in the presence and in the absence of CD3^+^ T cells. CD19^+^ B cell expansions are dependent on SpA surface display, as neither Δ*spa* nor *spa*_ΔLPXTG_ mutant staphylococci could activate proliferation.

### Human B cell proliferation induced by purified SpA variants.

**FIG 5:**
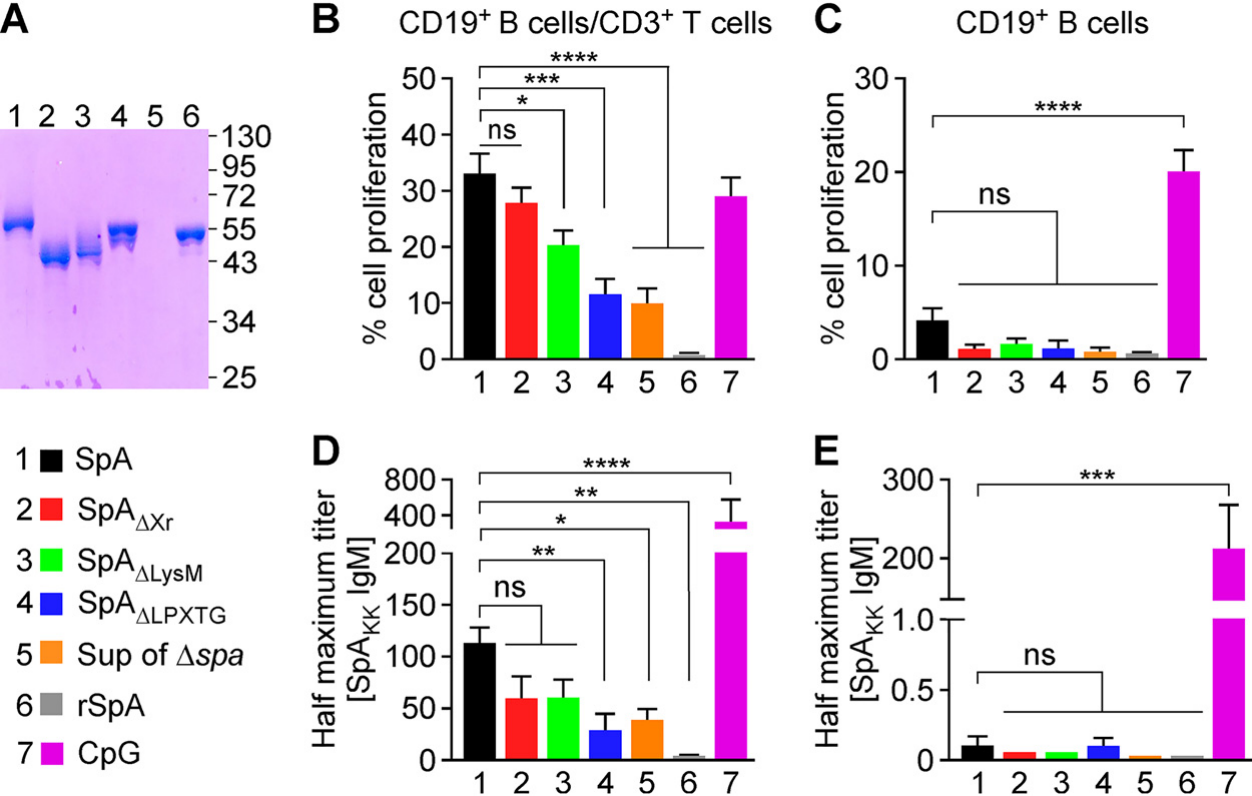
Human B cell proliferation stimulated by purified SpA and its variants. (A) Coomassie-stained SDS-PAGE of purified SpA and its variants from the supernatant of S. aureus cultures and of recombinant rSpA from E. coli. Numbers to the right of the gel indicate the sizes of molecular weight markers in kilodaltons. (B and C) Proliferation of CD19^+^ B cells stimulated by purified SpA and its variants (80 nM). CpG DNA was used as a positive control. (D and E) Secretion of IgM by B cells after stimulation with bacteria was analyzed by ELISA against SpA_KK_. For panels B and D, cells were isolated by negative selection (CD19^+^/CD3^+^). For panels C and E, cells were isolated by positive selection (CD19^+^). Data are the means (±SEM) of experimental replicates (*n *= 3 or 4) using B cells from different human donors. Statistical analysis was performed with one-way ANOVA with Dunnett’s multiple-comparison test. ****, *P < *0.0001; ***, *P < *0.001; **, *P < *0.01; *, *P < *0.05.

To determine whether SpA alone is sufficient to induce proliferation of human CD19^+^ B cell populations, full-length SpA and variants SpA_ΔXr_, SpA_ΔLysM_, and SpA_ΔLPXTG_ were purified from the filtered medium of S. aureus cultures via affinity chromatography on IgG Sepharose (Fig. 5A). Recombinant SpA (rSpA) (Fig. 1A) starting with six histidyl residues instead of the signal sequence and ending with the sorting motif followed by the Strep-tag sequence (LPETGWSHPQFEK) was purified from the cytosol of E. coli (Fig. 5A). Incubation of mixed populations of CD19^+^ B cells and CD3^+^ T cells with SpA caused proliferation of CD19^+^ B cells (32.46% ± 8.41%), while rSpA did not trigger B cell expansion (Fig. 5B). As a control, subjecting filtered, conditioned medium of centrifuged S. aureus Δ*spa* cultures to affinity chromatography generated a sample that induced proliferation of 9.80% ± 5.37% of CD19^+^ B cells in the mixed CD19^+^ B cell/CD3^+^ T cell populations (Fig. 5B, Δ*spa* mutant versus rSpA, *P = *0.01). We surmise that the mitogenic activity associated with the Δ*spa* sample may be due to lipoprotein or nucleic acid contamination and activation of Toll-like receptors (TLRs) (27). Incubation of CD19^+^ B cell/CD3^+^ T cell populations with nonmethylated CpG, a potent inducer of TLR9 signaling (27), induced the proliferation of 29.04% ± 5.68% CD19^+^ B cells (Fig. 5B). SpA_ΔXr_ induced CD19^+^ B cell proliferation similar to that of SpA, while removal of the LysM domain (SpA_ΔLysM_) and LPXTG sorting signal (SpA_ΔLPXTG_) led to a gradually reduced ability to promote B cell proliferation (Fig. 5B). In fact, SpA_ΔLPXTG_ activation was similar to that observed with Δ*spa* (Fig. 5B). Purified SpA molecules were also added to purified (97 to 99% pure) CD19^+^ B cells (Fig. 5C). Compared to CpG, SpA caused a modest CD19^+^ B cell proliferation (4.14% ± 2.46% versus 19.95% ± 4.03% [Fig. 5C]). None of the other protein preparations (SpA_ΔXr_, SpA_ΔLysM_, SpA_ΔLPXTG_, rSpA, and supernatant of Δ*spa*) induced the proliferation of purified CD19^+^ B cells (Fig. 5C). To rule out a contribution from contaminating lipopeptides to CD19^+^ B cell proliferation, SpA was also purified from the *lgt* mutant (28) (Fig. S3A). Lipoprotein diacylglycerol transferase (Lgt) catalyzes transfer of phosphatidylglycerol to the sulfhydryl moiety of a cysteine residue conserved in the signal peptides of lipoprotein precursors (29). This diacylglycerol modification modulates host immune responses (30) but is abrogated in the *lgt* mutant of S. aureus (28). Nonetheless, CD19^+^ B cell expansion by SpA was independent of diacylglycerol modification of lipoprotein precursors catalyzed by Lgt (Fig. S3B). As observed earlier, mitogen-active SpA induced CD19^+^ B cells to secrete IgM but only in the presence of CD3^+^ T cells (Fig. 5D and E). Incubation with rSpA did not promote IgM secretion, whereas CpG-activated CD19^+^ B cells secreted IgM regardless of CD3^+^ T cells (Fig. 5D and E). SpA_ΔXr_ and SpA_ΔLysM_ also activated IgM secretion in mixed CD19^+^ B cell/CD3^+^ T cell preparations, albeit to a lesser degree than wild-type SpA without significant difference (Fig. 5D). Similar lower levels of IgM secretion were observed when CD19^+^ B cells/CD3^+^ T cells were treated with SpA_ΔLPXTG_ or with the Δ*spa* control sample (Fig. 5D). Thus, SpA molecules are sufficient to promote proliferation of CD19^+^ B cells and secretion of V_H_3 idiotypic immunoglobulin in a CD4^+^ T helper cell-dependent manner. However, both the LysM domain and LPXTG sorting motif are required for optimal B cell superantigen activity.

10.1128/mBio.00039-21.4 FIG S3 Staphylococcal lipoproteins do not contribute to SpA-mediated stimulation of human B cells. (A) Coomassie-stained SDS-PAGE of SpA purified from the supernatant of cultures of wild-type strain Newman or the isogenic *lgt* mutant. Numbers to the left of the gel indicate the sizes of molecular weight markers in kilodaltons. (B) Proliferation of CD19^+^ B cells stimulated by purified SpA (80 nM). PBS was used as a negative control. CD19^+^ cells were isolated by positive selection (CD19^+^). Data are the means (±SEM) of experimental replicates (*n *= 3 or 4) using B cells from different human donors. Statistical analysis was performed with one-way ANOVA with Dunnett’s multiple-comparison test. **, *P < *0.01. Download FIG S3, DOCX file, 0.8 MB. Copyright © 2021 Shi et al. 2021 Shi et al. https://creativecommons.org/licenses/by/4.0/ This content is distributed under the terms of the Creative Commons Attribution 4.0 International license.

### Noncovalent peptidoglycan modification of released SpA.

Cell wall hydrolases release LPXTG anchored SpA from peptidoglycan with the C-terminal structure l-Ala-d-iGln-l-Lys(SpA-LPET-Gly_5_)-d-Ala-Gly_4_ (23). Earlier findings, and data presented here, suggest that this covalent modification is critical for the B cell superantigen activity of SpA (12). However, the LPXTG wall peptide modification does not account for the contribution of the predicted LysM domain. LysM domains have been described for proteins from bacteria, plants, and eukaryotes as independent folding units; in bacteria and plants, the LysM domain binds oligosaccharides comprised of GlcNAc and other amino sugar residues (31). To investigate the molecular basis for LysM domain-mediated B cell superantigen activity, purified SpA molecules released into the conditioned medium of cultures from S. aureus Newman and its *spa*_ΔLysM_ variants were cospotted with matrix and analyzed by matrix-assisted laser desorption ionization–time of flight mass spectrometry (MALDI-TOF MS), focusing on signals with *m/z* 700 to 4,000 in the positive ion mode (Table 1). These experiments identified two main ions, *m/z* 1,989.24 and *m/z* 3,554.07, detectable in wild-type SpA. *m/z* 1,989.24 was interpreted as the sodium ion of (MurNAc-GlcNAc)-(AQKG_5_A)-(MurNAc)-(AQKG_2_A) (calculated *m/z* 1,989.90). *m/z* 3,554 was interpreted as (MurNAc-GlcNAc)_2_-(AQKG_5_A_2_)_2_-(MurNAc-GlcNAc)-(AQKG_3_A) (predicted *m/z* 3,555.34). Several other ion signals, predicted as peptidoglycan fragments with glycan chains of 2 or 3 MurNAc-GlcNAc repeats and attached wall peptides, were also detected in wild-type SpA samples (Table 1). Importantly, none of the ion signals listed in Table 1 was observed for the SpA_ΔLysM_ sample. Taken together, these experiments suggest that released SpA copurifies with noncovalently bound peptidoglycan comprised of glycan chains that are 3 to 6 amino sugar residues in length. Copurification with glycan chains was not observed for the SpA_ΔLysM_ variant.

**TABLE 1:** MALDI-TOF analysis of released protein A from wild-type S. aureus Newman and its variant s*pa*_ΔLysM_

Proposed structure^*a*^	Predicted *m*/*z*	Observed *m*/*z*		
		WT^*b*^	Δ^*c*^	*spa*_ΔLysM_
(MurNAc-GlcNAc)-(AQKG_5_A)-(MurNAc)-(AQKA)	1,875.85	1,873.64	−2.21	Not observed
(MurNAc-GlcNAc)-(AQKG_5_A)-(MurNAc)-(AQKG_2_A)	1,989.90	1,989.24	−0.66	Not observed
(MurNAc-GlcNAc)_2_-(AQKG_2_A)_2_	2,021.90	2,021.08	−0.82	Not observed
(MurNAc-GlcNAc)-(AQKG_3_A_2_)-(MurNAc-GlcNAc)-(AQKA_2_)	2,106.95	2,107.05	0.10	Not observed
(MurNAc-GlcNAc)-(AQKG_5_A)-(MurNAc-GlcNAc)-(AQKG_2_A)	2,192.97	2,191.80	−1.17	Not observed
(OAc-MurNAc-GlcNAc)_2_-(AQKG_4_A)_2_	2,476.36	2,475.29	−1.07	Not observed
(OAc-MurNAc-GlcNAc)_2_-(AQKG_5_A_2_)_2_	2,732.48	2,732.28	−0.20	Not observed
(MurNAc-GlcNAc)_2_-(AQKA)_2_-(MurNAc-GlcNAc)-(AQKG_3_A_2_)	2,914.09	2,913.98	−0.11	Not observed
(MurNAc-GlcNAc)_2_-(AQKG_3_A)_2_-(MurNAc-GlcNAc)-(AQKA_2_)	3,085.16	3,085.14	−0.02	Not observed
(MurNAc-GlcNAc)_2_-(AQKG_5_A_2_)_2_-(MurNAc-GlcNAc)-(AQKG_3_A)	3,555.34	3,554.07	−1.27	Not observed

a Ion signals and proposed composition of compounds from MALDI-TOF mass spectra. Ions represent sodium adducts.

b WT, wild type.

c Δ, the difference between predicted and observed *m*/*z*.

### SpA LysM binds staphylococcal peptidoglycan.

**FIG 6:**
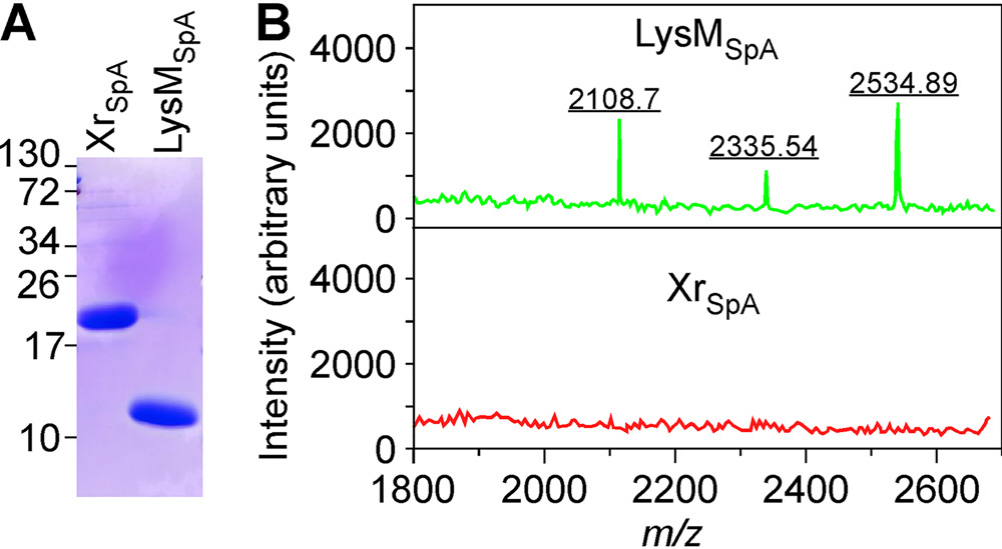
LysM_SpA_ binds staphylococcal peptidoglycan. (A) Coomassie-stained SDS-PAGE of purified Xr_SpA_ and LysM_SpA_. Numbers to the left of the gel indicate the sizes of molecular weight markers in kilodaltons. (B) Positive-ion MALDI-TOF mass spectra of lysostaphin-digested peptidoglycan eluting from LysM_SpA_ or Xr_SpA_ column. Ion signals represent sodium adducts.

To determine whether the LysM domain of SpA alone is sufficient to bind peptidoglycan fragments, recombinant LysM (LysM_SpA_) and region X (Xr_SpA_) with appended N- and C-terminal six-histidyl tags and Strep-tags were purified from the cytoplasm of E. coli via affinity chromatography (Fig. 6A). StrepTactin-Sepharose was loaded with purified protein, washed, and used for affinity chromatography of purified staphylococcal peptidoglycan solubilized with lysostaphin, which cleaves the pentaglycine cross bridges of peptidoglycan. Columns were washed and proteins with or without bound ligand were eluted with desthiobiotin, dialyzed against water, and analyzed by MALDI-TOF MS. Compounds with *m/z* 2,108.75, *m/z* 2,335.54 and *m/z* 2,534.89 were identified in the eluate of the LysM_SpA_-peptidoglycan copurification experiment (Fig. 6B). *m/z* 2,108.75 has the predicted structure (MurNAc-GlcNAc)-(AQKG_3_A_2_)-(MurNAc-GlcNAc) -(AQKA_2_) (calculated sodium ion *m/z* 2,106.95). The predicted structure of *m/z* 2,335.54 is (MurNAc-GlcNAc)-(AQKG_5_A_2_)-(MurNAc-GlcNAc)-(AQKG_2_A_2_) (calculated sodium ion *m/z* 2,335.04), whereas *m/z* 2,534.89 was designated (OAc-MurNAc-GlcNAc)-(AQKG_4_A_2_)-(OAc-MurNAc-GlcNAc)-(AQKG_5_A_2_) (calculated sodium ion *m/z* 2,533.38). Of note, monosaccharide (MurNAc)-(AQKG_3_A_2_) or disaccharide (MurNAc-GlcNAc)-(AQKG_3_A_2_) murein fragments, which are abundantly present in lysostaphin-digested peptidoglycan, were not retained during affinity chromatography on a LysM_SpA_ column (32). As a control, peptidoglycan fragments were not retained during chromatography on an Xr_SpA_ column (Fig. 6B). Thus, the LysM domain may capture glycan chains of peptidoglycan fragments released during S. aureus growth to implement the B cell superantigen activity of SpA together with the C-terminal peptidoglycan modification at its LPXTG motif.

### Recombinant rSpA modified with staphylococcal peptidoglycan displays mitogenic activity.

**FIG 7:**
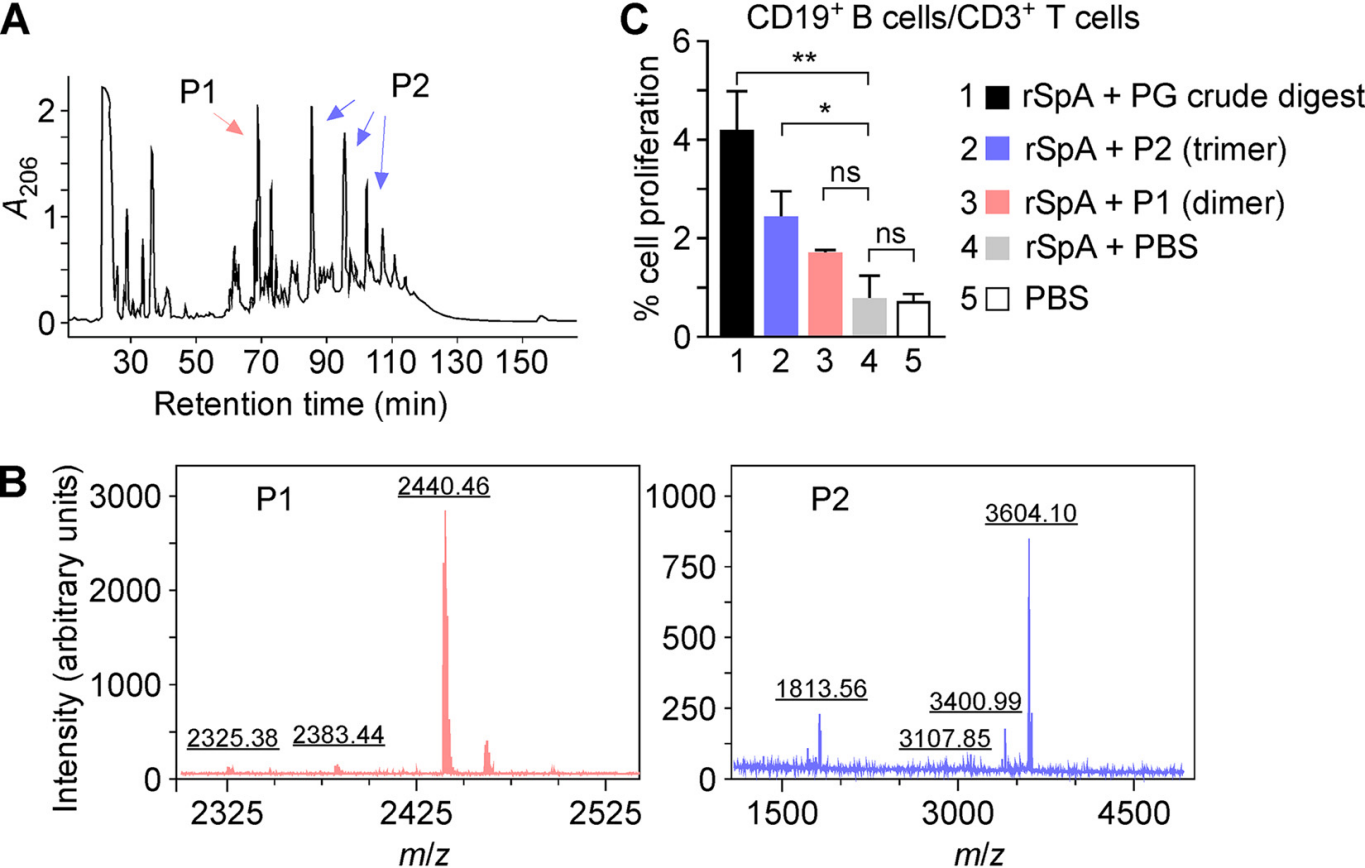
Human B cell proliferation stimulated by rSpA bound to peptidoglycan. (A) Peptidoglycan (5 mg) prepared from S. aureus Newman was treated with mutanolysin, and solubilized products were resolved by HPLC over a C_18_ column by measuring absorbance at 206 nm over time. (B) Positive-ion MALDI-TOF MS identification of peptidoglycan fragments from pooled fractions shown in panel A. Pink and blue arrows depict dimer and trimer, respectively, with confirmed *m/z* of 2,440.4 and 3,604.1. (C) Proliferation of negatively selected human B cells (CD19^+^/CD3^+^) stimulated by rSpA in PBS or rSpA preincubated with sortase A, crude peptidoglycan (PG) digest, trimer or dimer of PG as purified for panel A. Statistical analysis was performed with one-way ANOVA. **, *P < *0.01; *, *P < *0.05.

Previous studies demonstrated that a construct containing only the five IgBDs of SpA (5-IgBD) can bind V_H_3 idiotypic immunoglobulins and mediate cross-linking of BCRs on isolated human CD19^+^ B cells but cannot induce B cell expansion (12, 27, 33). When B cells were costimulated with the TLR2 agonist Pam3CSK4 or with the T cell cytokine interleukin 2 (IL-2), the 5-IgBD polypeptide promoted B cell proliferation but failed to promoted Ig production (27). rSpA used in this study was purified from the cytosol of E. coli and is not processed by leader peptidase or sortase A (Fig. 1A). Like the 5-IgBD construct (27), rSpA cannot induce B cell expansion, immunoglobulin gene expression, or IgM secretion (Fig. 4BD). rSpA with its intact LysM and sortase cleavage site (LPET/G) is an ideal tool to evaluate the contribution of peptidoglycan ligands. Purified peptidoglycan was treated with mutanolysin, and solubilized fragments were separated by reversed-phase high-performance liquid chromatography (rpHPLC) (Fig. 7A). Fractions eluting with increasing retention times were pooled and subjected to MALDI-TOF MS (Fig. 7A and B, P1 [pool 1] and P2). Peptidoglycan fragments with *m/z* 2,440.46 and 3,604.10 were identified as sodium ions of dimer (MurNAc-GlcNAc)-(AQKG_5_AA)-(MurNAc-GlcNAc)-(AQKG_5_A) (calculated *m/z* 2,440.68) and trimer (MurNAc-GlcNAc)_2_-(AQKG_5_A)_2_-(MurNAc-GlcNAc)-(AQKG_5_AA) (calculated *m/z* 3,604.97) (Fig. 7B). Of note, ion with *m/z* 1,813.56 corresponded to the doubly charged version of ion with *m/z* 3,604.10, and thus, only one compound from P2 could be identified by MALDI-TOF MS (Fig. 7B, right). rSpA was incubated with mutanolysin-treated peptidoglycan or purified dimer and trimer and sortase A. Following the removal of excess ligands and enzyme, rSpA preparations were dialyzed against buffer control and added to mixed populations of CD19^+^ B cells/CD3^+^ T cells. As expected, mock-treated rSpA resulted in negligible CD19^+^ B cell proliferation compared to that with buffer (0.80% ± 0.468% versus 0.69% ± 0.192%) (Fig. 7C). Incubation with rSpA exposed to mutanolysin-treated peptidoglycan, either the total peptidoglycan digest or pooled fractions containing peptidoglycan trimer, stimulated a small but significant proliferation of B cells (4.13% ± 0.834% and 2.39% ± 0.547%, respectively). Thus, peptidoglycan modification of rSpA results in a gain of mitogenic activity.

## DISCUSSION

During cell division, S. aureus forms septal membranes and synthesizes cross wall peptidoglycan with attached SpA at midcell (34, 35). Once cross wall assembly is completed, muralytic enzymes split the peptidoglycan layer and separate two spherical cells (21, 36–39). Muralytic enzyme cleavage at cross wall peptidoglycan also provides for the surface display of cell wall-anchored SpA and for the release of peptidoglycan-linked SpA into the extracellular medium (21, 23). Early work demonstrated that S. aureus stimulates the proliferation of isolated human B cells in a SpA-dependent manner (8, 9). SpA purified from S. aureus cultures promotes B cell proliferation in mice and in human lymphocyte preparations. B cell proliferation requires direct interaction of SpA with B cell receptors as well as CD4^+^ T helper cells (10–12). Recombinant SpA (rSpA) purified from E. coli does not promote B cell proliferation, which prompted a model whereby the peptidoglycan-modified LPXTG motif must be critical for the B cell superantigen activity of SpA. In this study, we validated this model by generating an S. aureus strain producing SpA lacking its C-terminal cell wall tethering motif, SpA_ΔLPXTG_. We completed our analysis by generating strains that produce SpA lacking the C-terminal Xr and LysM domain, SpA_ΔXr_ and SpA_ΔLysM_, respectively. When used as a mitogen, SpA_ΔLysM_ displayed reduced activity on human B cells compared to those with SpA and SpA_ΔXr_. We propose that this loss of activity correlates with LysM-mediated binding of peptidoglycan glycan chains. Further, some B cell superantigen activity could be restored following incubation of E. coli purified rSpA with sortase A and muralytic enzyme-treated peptidoglycan.

T cell-independent proliferation of isolated human B cells has also been reported (27). However, such B cell expansion requires large numbers of staphylococci and Toll-like receptor 2 (TLR2) activation by bacterial lipoproteins (8, 27). Such T cell-independent proliferation of human B cells may be restricted to tissues with significant pathogen burden, for example, the draining lymph nodes of deep-seated abscess lesions, where SpA cross-linking of B cell receptors may trigger staphylococcal uptake and TLR2 activation (40, 41). In contrast, localized S. aureus infection with low pathogen burden triggers systemic B cell proliferation and secretion of V_H_3 idiotypic immunoglobulin in humans (14). We propose that systemic modification of adaptive immune responses is associated with the release of SpA from the bacterial surface with two peptidoglycan modifications, wall peptides linked to the C-terminal LPXTG motif and glycan chains bound to the LysM domain. Released SpA molecules cross-link V_H_3 idiotypic BCRs to activate B cell proliferation and immunoglobulin secretion; however, this cannot be optimally accomplished without CD4^+^ T helper cells. Also required is receptor-interacting serine/threonine protein kinase 2 (RIPK2), presumably by implementing peptidoglycan signals via the nucleotide oligomerization domain 1 (NOD1) and 2 (NOD2) receptors (12, 42, 43). As NOD1 responds to muramyl peptide activation and NOD2 to wall peptides, it seems plausible that these two receptors may also be involved in perceiving peptidoglycan signals associated with SpA. Biotinylated peptidoglycan of S. aureus digested with mutanolysin has been shown to colocalize with NOD2 in primary mouse keratinocytes (44). It is unknown whether the mammalian immune system recognizes the ubiquitous polymeric glycan backbone of peptidoglycan via NOD2 or other receptors. Similarity searches show that humans and most mammals are endowed with the evolutionarily conserved proteins LysMD1, LysMD2, LysMD3, and LysMD4 with an N-terminal lysin motif (LysM). Only LysMD3 has been studied in mice. Mouse LysMD3 is a type II integral membrane protein that colocalizes with the Golgi apparatus (45). mLysMD3-deficient mice exhibit no obvious immune response deficiencies in a number of models of infection and inflammation (45). LysM domains have been best characterized for prokaryotes, bacteriophages, and plants. LysM domains are approximately 50 amino acids long, fold with a βααβ structure, and bind to GlcNAc-containing glycans, such as chitin, chitin-like compounds, and peptidoglycan (46). In bacteriophages and bacteria, LysM domains are mostly associated with catalytic protein modules for peptidoglycan assembly and degradation (22, 46). In plants, membrane proteins with multiple LysM modules mediate immune responses to bacterial pathogens as well as symbiotic recognition of bacteria (47, 48).

Future studies need to determine how B cells perceive the peptidoglycan signals of SpA, for example, following endocytosis of BCRs with cross-linked SpA, and to define the specific contributions of T cells to B cell proliferation and immunoglobulin secretion. In summary, this work provides insight into the mechanisms whereby localized infection with S. aureus and release of peptidoglycan-modified SpA trigger systemic B cell responses that divert adaptive immune responses to prevent the establishment of protective immunity.

## MATERIALS AND METHODS

### Bacterial strains and growth.

S. aureus strains Newman and RN4220 and variants were grown in tryptic soy broth (TSB) or on tryptic soy agar (TSA) at 37°C. Escherichia coli strains DH5α and BL21(DE3) were grown in Luria-Bertani broth (LB) or agar at 37°C. Ampicillin (100 μg/ml) and chloramphenicol (10 μg/ml) were used for the selection of pET-15b and pKOR1 derivatives in E. coli and S. aureus, respectively (49). Erythromycin (10 μg/ml) was used to select for *ermB*-marked mutations in S. aureus (50). Additional cloning procedures for the generation of *spa* strain variants are provided in the supplemental material, along with methods to examine the production and localization of SpA in the envelope of bacterial cells (Text S1; Table S1).

10.1128/mBio.00039-21.1 TEXT S1 Supplemental methods. Download Text S1, DOCX file, 0.05 MB. Copyright © 2021 Shi et al. 2021 Shi et al. https://creativecommons.org/licenses/by/4.0/ This content is distributed under the terms of the Creative Commons Attribution 4.0 International license.

### Animal experiments.

Animal experiments were conducted in accordance with institutional and federal guidelines following experimental protocol review and approval and supervision by the Institutional Biosafety Committee (IBC) and the Institutional Animal Care and Use Committee (IACUC) at the University of Chicago. Overnight cultures of bacteria were diluted 1:100 into fresh TSB and grown at 37°C with rotation to an *A*_600_ of 0.4. Staphylococci were sedimented by centrifugation at 13,000 × *g* for 5 min, washed, and suspended in PBS to an *A*_600_ of 0.4. Staphylococcal challenge doses (0.1-ml suspensions of bacteria in PBS) were quantified by spreading of sample aliquots on TSA plates, incubation, and enumeration of CFU. Cohorts (*n *= 10) of 6-week-old BALB/c mice were anesthetized via intraperitoneal injection of anesthetic (64.2 mg/ml of ketamine and 4.9 mg/ml of xylazine per kg of body weight). Anesthetized mice were infected by injection of 0.1 ml of 1 × 10^8^ CFU S. aureus into the periorbital venous sinus of the right eye. Mice were anesthetized and bled with heparin-coated capillary tubes from periorbital venous sinuses on days 5, 12, and 19 or by terminal cardiac puncture (day 26). Coagulated blood specimens were centrifuged at 10,000 × *g*; serum supernatant was retrieved for measurements of V_H_3 clonal IgG and IgM contents using ELISA (see Text S1 for details on ELISA). To ensure reproducibility, mouse experiments were repeated.

### Preparation of test articles used in B cell proliferation assays.

Details for B cell isolation and proliferation assays are provided in the supplemental material (Text S1). To examine the effect of killed bacteria on B cell proliferation, overnight cultures of S. aureus were diluted 1:100 into fresh TSB and grown at 37°C to an *A*_600_ of 0.4. For *itet-spa* and *itet-spa*_ΔLysM_ strains, 80 ng/ml and 400 ng/ml of anhydrotetracycline were added to cultures, respectively. Staphylococci were centrifuged, washed, and normalized to a load of 4 × 10^8^ CFU/ml. Bacteria were killed by addition of 0.5% formaldehyde and incubation at room temperature for 3 h, followed by heat treatment at 80°C for 3 min (9). Killed staphylococci were centrifuged for 5 min at 13,000 × *g*, the supernatant was discarded, and bacteria were suspended in the same volume of PBS. To examine the effect of purified SpA on B cell proliferation, first the *sbi*::*erm* allele was crossed in each background, yielding S. aureus *sbi*::*ermB* (isogenic wild type lacking Sbi), *spa*_ΔXr_ *sbi*::*ermB*, *spa*_ΔLysM_ *sbi*::*ermB*, *spa*_ΔLPXTG_ *sbi*::*ermB*, and Δ*spa sbi*::*ermB* variants, which were grown overnight in TSB and diluted 1:100 into 8 liters of fresh TSB. Cultures were grown with rotation at 37°C for 3 h and centrifuged at 8,000 × *g* for 10 min. Culture supernatants were filtered through 0.22-μm membranes (Millipore) and subjected to affinity chromatography over 2 ml of IgG Sepharose 6 Fast Flow (GE Healthcare). Bound proteins were eluted with 0.1 M glycine-HCl (pH 3.0), followed by immediate pH neutralization with 1 M Tris-HCl (pH 8.0). Eluates were concentrated in a vacuum centrifuge concentrator, dialyzed against PBS, and used at a concentration of 80 nM in B cell proliferation assays. Protein concentrations were determined with the Pierce bicinchoninic acid (BCA) protein assay kit (Thermo Fisher Scientific).

### Purification of peptidoglycan and recombinant proteins and binding experiments.

Peptidoglycan was prepared as described previously (51), suspended in water, normalized to an *A*_600_ of 10, and stored at −20°C. Recombinant proteins were produced using E. coli BL21(DE3) from crude lysate generated as described previously (26, 51, 52). Additional details are provided in the supplemental material (Text S1).

### Statistical analysis.

Statistical analysis was performed with GraphPad Prism (GraphPad Software, Inc., La Jolla, CA; version 7.04). Statistically significant differences were calculated by using statistical methods as indicated. *P* values of ≤0.05 were considered significant.

10.1128/mBio.00039-21.5 TABLE S1 Oligonucleotides used in this study. Download Table S1, DOCX file, 0.03 MB. Copyright © 2021 Shi et al. 2021 Shi et al. https://creativecommons.org/licenses/by/4.0/ This content is distributed under the terms of the Creative Commons Attribution 4.0 International license.
