# Supplementary material for: Peptidoglycan Contribution to the B Cell Superantigen Activity of Staphylococcal Protein A
Source: mBio. 2021 Apr 20;12(2):e00039-21. doi: 10.1128/mBio.00039-21 (PMC8092194; doi:10.1128/mBio.00039-21)
Supplement: FIG S1 [file mBio.00039-21-sf001.docx]

**FIG S1**


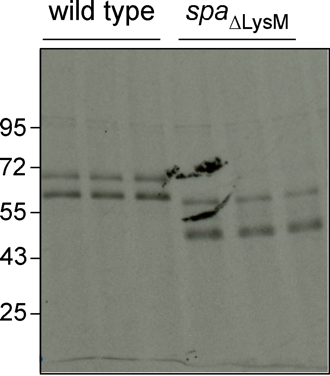


**Fig. S1.** **Rate of synthesis of SpA and SpA_ΔLysM_.** *S. aureus* cultures were pulse-labeled for 60 s with [35S]methionine/cysteine. Labeling was quenched by adding an excess of non-radioactive amino acids (chase) for 1 minute. After the chase, culture aliquots were precipitated with TCA, lysostaphin-treated, immunoprecipitated with αSpA and analyzed by autoradiography. The experiment was performed in triplicate. Numbers to the left of autoradiogram indicate the size of molecular weight markers in kDa. The slower and faster migrating species correspond to precursor and mature SpA and SpA_ΔLysM_, respectively. Owing to the truncation, precursor and mature SpA_ΔLysM_ migrate fatser than full length SpA.
