# Supplementary material for: Peptidoglycan Contribution to the B Cell Superantigen Activity of Staphylococcal Protein A
Source: mBio. 2021 Apr 20;12(2):e00039-21. doi: 10.1128/mBio.00039-21 (PMC8092194; doi:10.1128/mBio.00039-21)
Supplement: FIG S2 [file mBio.00039-21-sf002.docx]

**FIG S2**


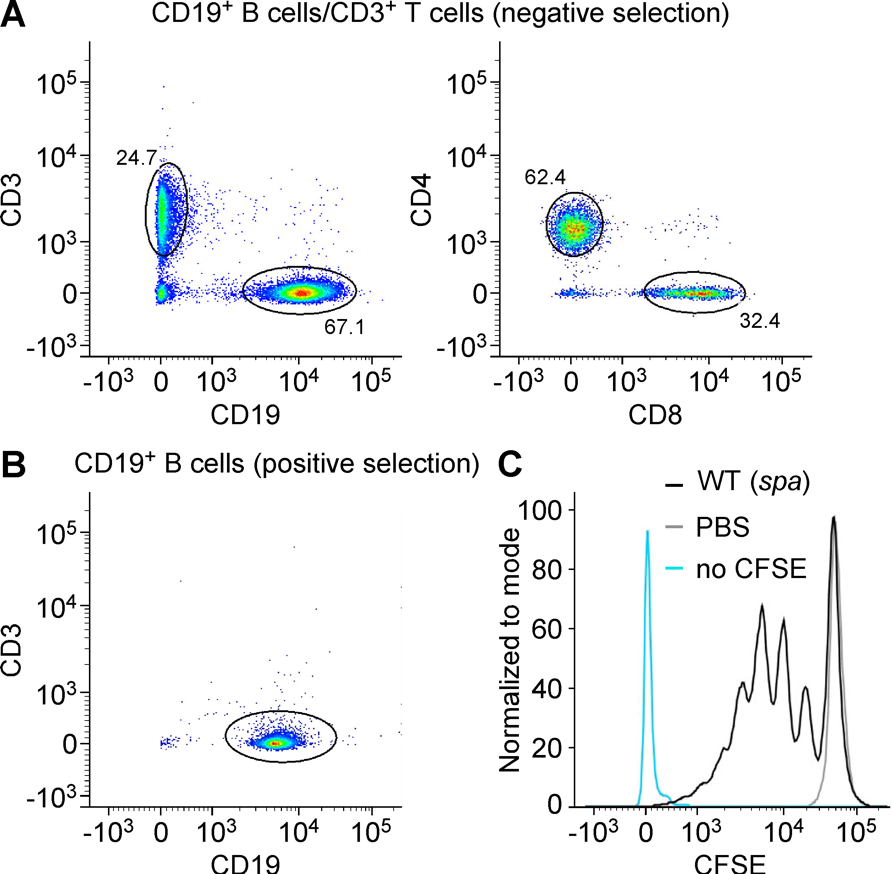


**Fig. S2.** **CD4^+^/CD8^+^ T lymphocyte ratio in human B cell preparations.** (**A**-**B**) Populations of CD19^+^ B cells and CD3^+^ T cells were quantified following depletion of human blood mononuclear cells with biotinylated antibodies against CD2, CD14, CD16, CD36, CD43 and CD235a and anti-biotin microbeads and exclusion of dead cells via Hoechst 33258 staining. Dead cells were excluded with Hoechst 33258 staining. (**C**) Histogram plot of the representative CFSE fluorescence of CD19^+^ B cell population after 6-day stimulation with killed *S. aureus*. Mock (PBS) treated B cells did not dilute CFSE signals (undivided population in grey), whereas *S. aureus* WT induced 1, 2, 3, 4 or 5 cell divisions (black) after 6 days. Proliferation in percent was calculated with (*i* is the division number, N*_i_* is the cell number in division *i*). Autofluorescent unlabeled CD19^+^ B cells are traced in blue.

$${\sum_{1}^{i} \frac{Ni}{2i}}/{\sum_{0}^{i} \frac{Ni}{2i}}$$
