# Supplementary material for: Peptidoglycan Contribution to the B Cell Superantigen Activity of Staphylococcal Protein A
Source: mBio. 2021 Apr 20;12(2):e00039-21. doi: 10.1128/mBio.00039-21 (PMC8092194; doi:10.1128/mBio.00039-21)
Supplement: FIG S3 [file mBio.00039-21-sf003.docx]

**FIG S3**


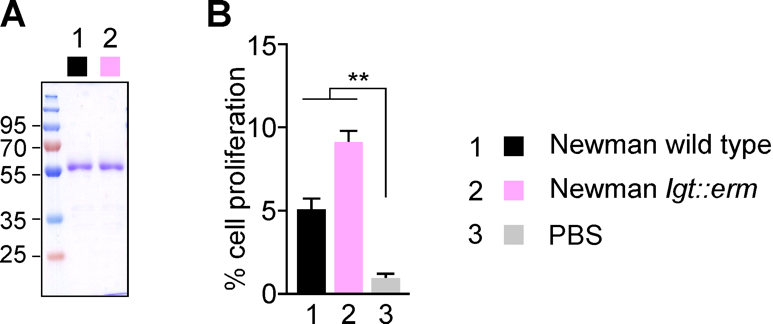


**Fig. S3.** **Staphylococcal lipoproteins do not contribute to SpA-mediated stimulation of human B cells.** (A) Coomassie-stained SDS-PAGE of SpA purified from the supernatant of cultures of wild type strain Newman or the isogenic *lgt* mutant. Numbers to the left of gel indicate the size of molecular weight markers in kDa. (**B**) Proliferation of CD19^+^ B cells (in percent) stimulated by purified SpA (80 nM). PBS was used as a negative control. CD19^+^ cells were isolated by positive selection (CD19^+^). Data are the mean (±SEM) of experimental replicates (*n*=3-4) using B cells from different human donors. Statistical analysis was performed with one-way ANOVA with Dunnett’s multiple comparisons test: **, *P*<0.01.
