# Supplementary material for: Peptidoglycan Contribution to the B Cell Superantigen Activity of Staphylococcal Protein A
Source: mBio. 2021 Apr 20;12(2):e00039-21. doi: 10.1128/mBio.00039-21 (PMC8092194; doi:10.1128/mBio.00039-21)
Supplement: TEXT S1 [file mBio.00039-21-s0001.docx]

**Supplemental Material**

**Peptidoglycan contribution to the B cell superantigen activity of Staphylococcal protein A**

Miaomiao Shi^a^, Stephanie E. Willing^b^, Hwan Keun Kim^b*^, Olaf Schneewind^(†)^, and Dominique Missiakas^a,b#^

^a^Howard Taylor Ricketts Laboratory, Argonne National Laboratory, Lemont, IL 60439; ^b^Department of Microbiology, University of Chicago, Chicago, IL 60637

* Present address: Department of Microbiology and Immunology, Stony Brook University, Stony Brook, NY.

^†^ Deceased 26 May 2019

^#^Address for correspondence: Howard T. Ricketts Laboratory, Argonne National Laboratory Building 204, 9700 South Cass Avenue, Argonne, IL 60439. Email [dmissiak@bsd.uchicago.edu](mailto:dmissiak@bsd.uchicago.edu)

**Running Title:** The LysM domain of Staphylococcal protein A

**Text S1**

**Supplemental Methods**

**Cloning and strain variants**. Primers used in this study are listed in Table S1. To generate *spa*_ΔXr_ mutation, primer pairs Xr-1/Xr-2 and Xr-3/Xr-4 were used to amplify the 1kb DNA sequence segments upstream and downstream of Xr domain from chromosome DNA of *S. aureus* Newman, respectively. Similarly, primer pairs LysM-1/LysM-2 and LysM-3/LysM-4 and primer pairs Xr-1/LPXTG-1 and LPXTG-2/Xr-4 were used to construct the deletion of LysM domain and LPXTG motif within *spa*, respectively. The two PCR products were ligated and cloned into pKOR1 using the Gateway BP Clonase II enzyme (INVITROGEN)(1). Derivatives of pKOR1 were electroporated first into the *S. aureus* RN4220, colonies formed at 30°C, plasmid were purified and electroporated into *S. aureus* Newman for colony formation at 30°C. Transformed staphylococci were then cultured at 42°C to block plasmid replication and promote recombination with chromosomal DNA. Colonies representing plasmid integrants were propagated at 30°C on TSA plate with 200 ng/ml anhydrotetracycline to promote allelic replacement and loss of pKOR1, generating the alleles *spa*_ΔXr_, *spa*_ΔLysM_, *spa*_ΔLPXTG_. Mutations were validated by PCR amplification of the mutated allele and DNA sequencing. The *sbi*::*ermB* allele was transduced with bacteriophage φ85 into *S. aureus* Newman and its *spa* variant strains (2). Primer pairs Xr_SpA_-1/Xr_SpA_-2, LysM_SpA_-1/ LysM_SpA_-2, SrtA-1/SrtA-2, rSpA-1/rSpA-2 were used to amplify the coding DNA for Xr_SpA_, LysM_SpA_, sortase A and SpA from *S. aureus* Newman chromosomal template. The downstream primers used to amplify these PCR products were designed to append the Strep-tag II peptide (WSHPQFEK) at the C-terminus of the polypeptides (Table S1). PCR products were cut with NdeI and BamHI or XhoI and ligated into pET-15b cut with the same restriction enzymes. As a result, the translated products or recombinant Xr_SpA_, LysM_SpA_, sortase A and SpA are modified with the sequence MGSSH_6_SSGLVPRGSH at the N-terminal. Resulting plasmids were transformed into *E. coli* DH5α, colonies isolated, plasmids purified and validated by DNA sequencing and transformed into *E. coli* BL21 (DE3) for expression and affinity purification of recombinant proteins.

**SpA localization in *S. aureus* and immunofluorescence microscopy**. Overnight cultures of *S. aureus* were diluted with 1:100 into fresh TSB and grown with rotation to an absorbance of 0.7 at 600 nm (*A*_600_). To analyze proteins in the whole culture, 75 µl of trichloroacetic acid (TCA; 100%) was added to 1 ml of culture. The sample was put on ice for 30 min and spun at 13,000 ×*g* for 10 min. Precipitated proteins and staphylococci were suspended in 1 ml 100 mM Tris-HCl (pH 7.0) supplemented with 50 μg/ml lysostaphin and incubated for 60 min with vigorous vortex to degrade the bacterial cell wall. Proteins were again precipitated with TCA. To examine the subcellular localization of SpA variants, 1 ml *S. aureus* culture aliquot was centrifuged at 13,000 ×*g* for 5 min. The culture supernatant (S) was separated from the bacterial sediment (P, pellet), transferred to another Eppendorf tube and proteins precipitated with TCA. Staphylococcal pellets were suspended in 1 ml TSM buffer [100 mM Tris-HCl (pH 7.0), 0.5 M sucrose, 10 mM MgCl_2_] supplemented with 50 μg/ml lysostaphin and incubated for 30 min at 37°C. The resulting protoplasts were sedimented by centrifugation at 13,000 ×*g* for 10 min. Proteins in the cell wall extract (W) were transferred with the supernatant to another Eppendorf tube and precipitated with TCA. Protoplasts were suspended in 1 ml TM buffer [100 mM Tris-HCl (pH 7.0) and 10 mM MgCl_2_] and lysed with three freeze-thaw cycles in dry-ice/ethanol and water baths. Membranes (M) were separated from cytoplasmic fractions (C) by centrifugation at 100,000 ×*g* for 40 min. Cytoplasmic proteins (C) in the supernatant were transferred to a new Eppendorf tube and precipitated with TCA. Membrane proteins (M) in the sediment were suspended in 1 ml TM and precipitated with TCA. All TCA precipitated proteins were washed in 1 ml ice-cold acetone for 10 min, dried and suspended in 50 μl 0.5 M Tris-HCl (pH 7.0), 4% SDS. Protein samples were mixed with an equal volume of 2× sample buffer, heated for 10 min at 90°C, separated on 12% SDS-PAGE, electrotransferred to PVDF membrane and subjected to immunoblot analysis with HRP-conjugated IgM for the detection of SpA. Rabbit polyclonal sera raised against sortase A (α-SrtA), secreted coagulase (α-Coa) and cytoplasmic ribosomal protein L6 (α-L6) were used as controls. Immuno-reactive signals were detected by HRP-conjugated anti-rabbit secondary IgG. For immunofluorescence microscopy, overnight cultures were diluted 1:100 into fresh TSB and grown at 37°C to *A*_600_ 0.7. Bacteria were centrifuged, washed in PBS, and fixed with 2.5% paraformaldehyde, 0.006% glutaraldehyde in 30 mM sodium phosphate (pH 7.4). Bacteria were incubated with anti-SpA_KKAA_ mouse antibody 3F6 for 1 hour, washed, incubated with Alexa Fluor 647-conjugated goat anti-mouse IgG (Invitrogen), washed with 3% PBS twice and PBS once and suspended in PBS (3). Bacteria were then incubated with 1 μg/ml vancomycin-BODIPY conjugate (Invitrogen) and 0.1 μg/ml DAPI for the staining of peptidoglycan and DNA, respectively. Aliquots (10 μl) of stained bacteria were pipetted onto coverslips for polylysine-treatment (10 min), mounted on glass slides containing one drop of SlowFade anti-fading reagent (Invitrogen) and sealed with nail polish. Images were captured through a Leica SP5 tandem-scanner spectral two-photon confocal microscope at the University of Chicago Light Microscopy Core Facility and analyzed with ImageJ.

**Immunoprecipitation.** Staphylococcal cultures were grown to *A*_600_ of 0.8 in TSB and bacteria were centrifuged at 5,000 ×*g* for 5 min. Bacterial pellets were washed twice and resuspended in 1 ml minimal medium IV lacking methionine and cysteine. [^35^S]methionine/cysteine (1 mCi/ml Perkin Elmer) was added to bacterial suspensions for 60 seconds at 37°C. 50 µl of Chase solution (2 mg/ml methionine, 2 mg/ml cysteine, 10 mg/ml casamino acids) was added to quench the reaction for 1 min. 250 µl bacterial suspension was removed and mixed with 250 µl ice-cold 10% TCA. Precipitates were spun at 13,000 ×*g* for 10 min and resuspended in 1 ml 0.5 M Tris-HCl (pH 7.0) containing 20 µg/ml lysostaphin for 1 hour at 37°C. Cell lysates were precipitated with 7% TCA, washed with acetone, dried, suspended in 50 µl 4% SDS, 0.5 M Tris-HCl (pH 7.5) and allowed to solubilize for 30 min prior to boiling. Next, samples were incubated for 1 hour with anti-SpA_KKAA_ mouse antibody 3F6 (3) in 1 ml RIPA buffer (0.1% SDS, 0.5% deoxycholic acid, 1% Triton X-100, 50 mM Tris-HCl pH 8.0, 150 mM NaCl). Protein A sepharose (50 µl of 50% slurry, Sigma) was added to each sample to purify immune complexes for 1 hour, followed by five washes with 1 ml RIPA buffer. Proteins bound to the beads were solubilized by boiling in 15 µl 2× sample buffer for 10 min and separated on 10% SDS-PAGE. Gels were dried on Whatman 3 M paper and autoradiographed on X-ray film for 2 more weeks in the dark room.

**Enzyme-linked immunosorbent assay (ELISA)**. To quantify V_H_3 clonal IgG and IgM in serum samples, purified recombinant SpA_KK_ [1 μg/ml SpA_KK_ in 0.1 M sodium carbonate buffer (pH 9.6)] was used to coat NUNC MaxiSorp ELISA plates (THERMOFISHER SCIENTIFIC) at 4°C overnight (4). The following day, plates were blocked with 5% (w/v) non-fat dry milk in PBS with 0.05% tween-20 and incubated with serially diluted mouse sera. Plates were incubated with HRP-conjugated secondary anti-mouse IgM and IgG antibodies and developed using OptEIA reagent (BD Biosciences). Half-maximal titers of V_H_3 clonal IgG and IgM were calculated using the GraphPad Prism Software.

**B cell isolation and proliferation assays**. Experiments with blood from human volunteers were performed with a protocol reviewed, approved and supervised by the University of Chicago's Institutional Review Board (IRB). Peripheral blood mononuclear cells (PBMC) were prepared by diluting human venous blood 4× into PBS (pH 7.2), 2 mM EDTA. Diluted blood cells (34 ml) were layered over 16 ml Ficoll-Paque^TM^ (MILTENYI BIOTECH) and centrifuged for 10 min at 1,000 ×*g* in Leucosep® tubes (GREINER). The mononuclear cell layer was retrieved, transferred to 50 ml conical tubes, mixed with 50 ml PBS (pH 7.2), 2 mM EDTA and centrifuged 300 ×*g* for 10 min at 20°C. Supernatant was removed, sedimented cells were suspended in 50 ml PBS (pH 7.2), 2 mM EDTA and centrifuged 200 ×*g* for 10 min at 20 °C. Platelets were removed with the supernatant and two additional sedimentation steps (200 ×*g* for 10 min at 20°C). PBMC were suspended in 10 ml PBS (pH 7.2), 2 mM EDTA subjected to the Dead Cell Removal Kit (MILTENYI BIOTECH) to avoid non-specific binding of dead cells to MACS Mirocrobeads. Live cells were subjected to the Human B Cell Isolation Kit II (MILTENYI BIOTECH), depleting non-B cells with a cocktail of biotinylated antibodies against CD2, CD14, CD16, CD36, CD43 and CD235a and Anti-Biotin Microbeads. Alternatively, CD19 MicroBeads (MILTENYI BIOTECH) were used for positive selection of human B cells (pro-B cells through B cell lymphoblasts) on MACS columns (MILTENYI BIOTECH). Isolated B cells were enumerated using a hemocytometer. Portions of cells were set aside to analyze non-stimulated B cells. Purified B cells were suspended in 1 ml PBS with 5% (v/v) HI-FBS. B cell preparations were treated with 5 μM carboxyfluorescein succinimidyl ester (THERMOFISHER, CellTrace^TM^ CFSE Cell Proliferation Kit) for 5 min and washed twice with 10 volumes of PBS containing 5% (v/v) HI-FBS. B cells were suspended in an appropriate volume of IMDM (Iscove's Modified Dulbecco's Medium from Gibco) supplemented with 10% (v/v) heat inactivated fetal bovine serum (HI-FBS, GIBCO), antibiotics (100 mg/ml penicillin and 100 mg/ml streptomycin), 2 mM L-glutamine and 0.1 mM β-mercaptoethanol at a final concentration of 5×10^5^ cells/ml. Aliquots of unlabeled and CFSE-labeled cells were added to 96-well plates where individual wells contained 4 μl test article and 200 μl cell suspension. Test articles included suspensions of killed staphylococci (added at a ratio of 16 bacteria per lymphocyte), 80 nM purified protein (SpA and its variants), and CpG DNA as a positive control. Cells were incubated at 37°C in a 5% CO_2_ milieu for 6 days, followed by flow cytometry analysis.

**Flow cytometry.** To examine B cell preparations, isolated B cells were stained with human α-CD19-PE (MILTENYI BIOTEC), α-APC-Cy7-CD3 (BD BIOSCIENCES), α-PerCP-Cy5.5-CD4 (BIOLEGEND), α-PE-Cy7-CD8 (BD BIOSCIENCES) and Hoechst 33258 (LIFE TECHNOLOGIES) and analyzed on LSRII 3-8 with the FACSDiva Software. PBMC were stained with the same markers to analyze compensation of spectral overlap in multicolor analysis. Data were analyzed with FlowJo Software. To measure B cell proliferation, stimulated cells were centrifuged at 300 ×*g* for 10 min. Supernatant was removed and transferred to another Eppendorf tube for measurements of IgM secretion. Cells were suspended in 100 μl FACS buffer [PBS (pH 7.2), 2 mM EDTA and 0.5% BSA] and stained with α-PE-CD19 and Hoechst 33258 in order to discriminate dead from viable cells. Cells were transferred into polystyrene round-bottomed tubes and subjected to flow cytometry analysis on a LSRII3-8 instrument (BD Biosciences) with suitable settings to exclude dead cells and select for B cells. The same settings were used for all samples in this experiment and data were analyzed by FlowJo Software.

**Purification of peptidoglycan and recombinant proteins and binding experiments.** Purified peptidoglycan (5 mg) was digested by overnight incubation at 37°C with either 0.1 mg Lysostaphin (AMBI PRODUCTS) in 1 ml 50 mM Tris-HCl buffer (pH 7.5) or with 10 μl mutanolysin (0.5 unit) in 1 ml 50 mM phosphate buffer, pH 5.5. The sample was centrifuged for 10 min at 8,000 ×*g*. Solubilized peptidoglycan fragments in the supernatant were passed over a Sep-Pak C18 Cartridge and eluted in 5 ml of 50 mM Tris-HCl buffer (pH 7.5). Recombinant LysM_SpA_, Xr_SpA_, rSpa and sortase A carrying N-terminal 6 histidyl and C-terminal Strep-tag II sequences were produced using *E. coli* BL21 (DE3) and purified from crude lysates onto 1 ml nickel-nitrilotriacetic acid agarose according to the manufacturer’s recommendations (Ni-NTA agarose, QIAGEN) and as described (4-6). Proteins were eluted with imidazole and loaded onto 1 ml StrepTactin-Sepharose as described by the manufacturer (IBA LIFESCIENCES). Fractions containing purified protein were identified by separating sample aliquots on 15% SDS-PAGE and Coomassie Blue staining. Samples were dialyzed against Buffer A (50 mM Tris-HCl (pH 8.0), 150 mM NaCl, 1 mM EDTA) and stored at −20°C. Protein concentration was determined with the Pierce BCA Protein Assay Kit (THERMOFISHER SCIENTIFIC). For binding experiments with peptidoglycan, LysM_SpA_ and Xr_SpA_ purified proteins (0.5 mg) were loaded onto 1 ml StrepTactin-Sepharose columns. Columns were washed with 15 ml Buffer B (50 mM Tris-HCl pH 7.5, 150 mM NaCl) and loaded with 1 ml lysostaphin-digested peptidoglycan or mock [Buffer C, 1 ml 50 mM Tris-HCl (pH 7.5)]. Columns were washed with 50 ml Buffer B and eluted with 2.5 mM desthiobiotin. LysM_SpA_ and Xr_SpA_ eluates were dialyzed against water, dried under vacuum and suspended in 20 μl 0.1% TFA. Aliquots (1 μl) were co-spotted 1:1 with an equal volume of α-cyano-4-hydroxycinamic acid matrix or sinapic acid matrix onto MALDI-TOF sample plates and dried. Samples were analyzed in the reflector-positive mode with an Autoflex Speed Bruker MALDI-TOF mass spectrometry instrument pre-calibrated with known molecular weight standards. For peptidoglycan modification of rSpA, the purified protein (0.5 mg) was loaded onto 1 ml Ni-NTA agarose. 1 ml mutanolysin-digested peptidoglycan, purified dimer or trimer fragments of peptidoglycan or mock (Buffer C) was passed over the column along with sortase A (5 μM). Ni-NTA beads were washed with 15 ml Buffer C and proteins eluted with 0.5 M imidazole. Eluted fractions were re-purified over 1 ml StrepTactin-Sepharose. Sortase A and unprocessed rSpA are retained on this column. rSpA cleaved at the LPETG motif by sortase A eluted with the flow through and was dialyzed against Buffer C before addition to B cells at a concentration of 80 nM.

**Reference:**

1. Bae T, Schneewind O. 2005. Allelic replacement in *Staphylococcus aureus* with inducible counter-selection. Plasmid 55:58-63.

2. Bae T, Banger AK, Wallace A, Glass EM, Aslund F, Schneewind O, Missiakas DM. 2004. *Staphylococcus aureus* virulence genes identified by *bursa aurealis* mutagenesis and nematode killing. Proc Natl Acad Sci USA 101:12312-12317.

3. Kim HK, Emolo C, DeDent AC, Falugi F, Missiakas DM, Schneewind O. 2012. Protein A-specific monoclonal antibodies and the prevention of *Staphylococcus aureus* disease in mice. Infect Immun 80:3460-3470.

4. Falugi F, Kim HK, Missiakas DM, Schneewind O. 2013. The role of protein A in the evasion of host adaptive immune responses by *Staphylococcus aureus* mBio 4:e00575-13.

5. Chan YG, Frankel MB, Missiakas D, Schneewind O. 2016. SagB glucosaminidase Is a determinant of *Staphylococcus aureus* glycan chain length, antibiotic susceptibility, and protein secretion. J Bacteriol 198:1123-1136.

6. Bobrovskyy M, Willing SE, Schneewind O, Missiakas D. 2018. EssH Peptidoglycan Hydrolase Enables Staphylococcus aureus Type VII Secretion across the Bacterial Cell Wall Envelope. J Bacteriol 200.
