# Supplementary material for: Peptidoglycan Contribution to the B Cell Superantigen Activity of Staphylococcal Protein A
Source: mBio. 2021 Apr 20;12(2):e00039-21. doi: 10.1128/mBio.00039-21 (PMC8092194; doi:10.1128/mBio.00039-21)
Supplement: TABLE S1 [file mBio.00039-21-st001.docx]

| **Table S1. Oligonucleotides used in this study** | |
| --- | --- |
| Primer | Oligonucleotide sequence |
| Xr-1 | GGGGACAAGTTTGTACAAAAAAGCAGGCTGGTGTAGGTATTGCATCTGTAAC |
| Xr-2 | TACCATCTTCTGGTGCTTGAGCATCGTT |
| Xr-3 | TCAAGCACCAGAAGATGGTAACGGAGTACA |
| Xr-4 | GGGGACCACTTTGTACAAGAAAGCTGGGTTTGGACAAGGTATTGCTAAAG |
| LysM-1 | GGGGACAAGTTTGTACAAAAAAGCAGGCTTATGAAATCTTGAACATGCC |
| LysM-2 | GCTTCTTATCTCCGTTACCATCTTCTTTACC |
| LysM-3 | TGGTAACGGAGATAAGAAGCAACCAGCAAA |
| LysM-4 | GGGGACCACTTTGTACAAGAAAGCTGGGTGCAGGTGCTTTATTGGTC |
| LPXTG-1 | GTTTGTTTTTATGCTTGAGCTTTGTTAGCAT |
| LPXTG-2 | AGCTCAAGCATAAAAACAAACAATACACAACG |
| Xr_SpA_-1 | GGAATTCCATATGCAAGCACCAAAAGAGGAAGAC |
| Xr_SpA_-2 | CGGGATCCTTA**TTTTTCGAACTGCGGGTGGCTCCA**TCCGTTACCATCTTCTTTACCAG |
| LysM_SpA_-1 | GGAATTCCATATGGGTAACGGAGTACATGTCG |
| LysM_SpA_-2 | CGGGATCCTTA**TTTTTCGAACTGCGGGTGGCTCCA**CTTCTTATCAACAACAAG |
| SrtA-1  SrtA-2 | nnCATATGCAAGCTAAACCTCAAATTCCG  AACTCGAGTCA**CTTCTCAAATTGAGGATGAGACCA**TTTGACTTCTGTAGCTACAAAGATTTTACG |
| rSpA-1  rspA-2 | nnCATATGGCGCAACACGATGAAGCTCAACAAAATGC  CGGGATCC**CTTCTCAAATTGAGGATGAGACCA**ATGACCACCAGTTTCTGGTAATGCTTGAGCTTTGTTAGC |

Underlined nucleotides represent *attB* sequences for cloning into pKOR1. Bold sequences encode for the Strep-tag II peptide.
